# Online Coupling of Lab-on-Valve Format to Amperometry Based on Polyvinylpyrrolidone-Doped Carbon Paste Electrode and Its Application to the Analysis of Morin

**DOI:** 10.1155/2012/257109

**Published:** 2012-04-05

**Authors:** Yang Wang, Guojun Yao, Jie Tang, Chun Yang, Qin Xu, Xiaoya Hu

**Affiliations:** College of Chemistry and Chemical Engineering, Yangzhou University, Yangzhou 225002, China

## Abstract

The potential capabilities and analytical performance of lab-on-valve (LOV) manifold as a front end to amperometry have been explored for the on-line determination of morin. Meanwhile, the electrochemical behaviors of morin were investigated based on polyvinylpyrrolidone- (PVP-) doped carbon paste electrode (CPE), which found that PVP can significantly improve its oxidation peak current. The excellent amperometric current response was achieved when the potential difference (Δ*E*) of 0.6 V was implemented in pH 6.5 phosphate buffer solution (PBS) that served as the supporting electrolyte. A well-defined oxidation peak has been obtained in studies using PVP as a modifier of CPE based on the oxidation of morin. The present work introduces the LOV technique as a useful tool for amperometric measurement, documents advantages of using programmable flow, and outlines means for miniaturization of assays on the basis of PVP modified CPE. The proposed method was applied successfully to the determination of morin in real samples, and the spiked recoveries were satisfactory.

## 1. Introduction

**Figure 1:**
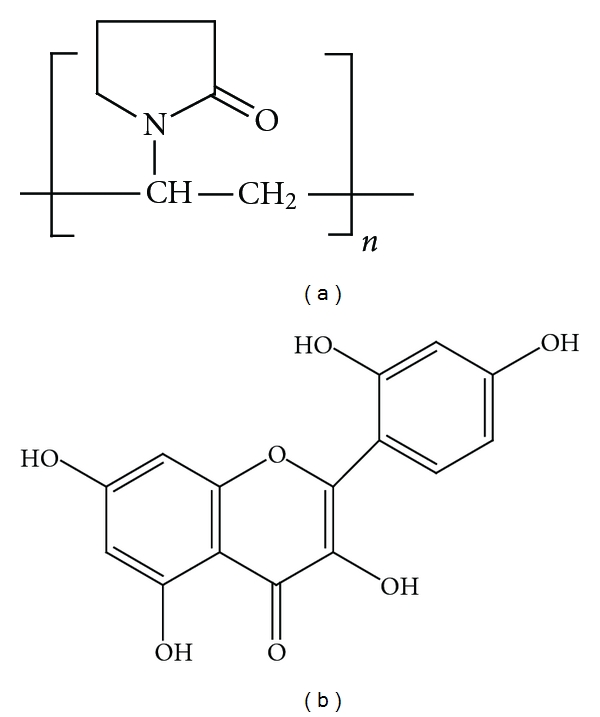
The structures of PVP (a) and morin (b).

As for polyvinylpyrrolidone (PVP) mentioned here, which is a water-soluble synthetic polymer, it is an amphiphilic high molecular-weight compound containing highly polar amide groups in conjunction with apolar methylene and methane (CH) moieties [1]. And its chemical structure is presented in Figure 1(a). PVP is a versatile polymer with innate surface activity and has the capability to form strong adsorption to phenolic compounds, which is attributed to hydrogen bonds between imide in the center of polymer and hydroxyl group in phenolic compounds [2]. With the above characteristics, PVP can furnish different electrical properties to the modified electrode/solution interface. The applications of carbon paste electrode (CPE) and chemically modified carbon paste electrode (CMCPE) in analytical chemistry have attracted considerable attention in recent years. In comparison with other modified electrodes, CMCPEs exhibit advantages such as ease of fabrication and regeneration, low background current, wide range of used potential, stable response, and very low Ohmic resistance [3–5].

Flavonoids are the most abundant polyphenolic compounds widely distributed in fruits, vegetables, and plant-derived beverages such as tea and red wine [6, 7]. Due to many reports claiming that flavonoids have a variety of beneficial pharmacological properties, including antioxidation, antiviral, and anti-inflammatory activities, flavonoids have recently been paid more attention as potential therapeutic agents [8–12]. Morin (2′,3,4′,5,7-pentahydroxyflavone), whose molecular structure is described in Figure 1(b), as a member of the family of the flavonols, is an important bioactive compound via interacting with nucleic acids, enzymes, and protein [13]. Furthermore, with the potent antioxidant and metal ion chelating capacity, morin possesses various biological and biochemical effects such as anti-inflammation, antineoplasm, and antimutagenesis [14–18]. Thus, it is necessary that reliable and sensitive analytical methods should be developed for the determination of morin.

 As far as we know, there is a few information available in the literatures concerning the electrochemical behavior of morin [11, 12, 19]. Tesio et al. studied the thermodynamic and kinetics of the adsorption of morin on glassy carbon (GC) electrodes by both cyclic and square wave voltammetries [20]. The Frumkin adsorption isotherm was the best to describe the specific interaction of morin with GC electrodes. Xiao et al. reported that a multiwalled carbon nanotubes-paraffin oil paste electrode was fabricated and used to study the voltammetric behavior of morin [21]. Morin exhibited a very sensitive and well-defined anodic peak on it.

In recent years, more attention was focused on the development of automated analytical procedures. And the development of sequential injection lab-on-valve (SI-LOV) format provides vast potentials for instrumental miniaturization and automatization and online detection [22–25]. The volumes and dimensions of fluidics are downscaled in a “digital” fashion by stopping, reversing, and accelerating flow rates, with the aim of making this technique compatible with advances in instrument miniaturization [26–28]. The present work adds a novel facet to the LOV technology for the determination of morin by implementing amperometric measurement. By means of integrating appropriate electrochemical flow cell (EFC) designed and processed into LOV unit, LOV-amperometric system was developed via the communication with electrodes, realizing automated online analysis in a closed system. The research effort is devoted to investigating the electrochemical properties of morin using PVP/CPE and the usefulness of the method was evaluated for the determination morin in real samples.

## 2. Experimental

### 2.1. Instrumentation

**Figure 2:**
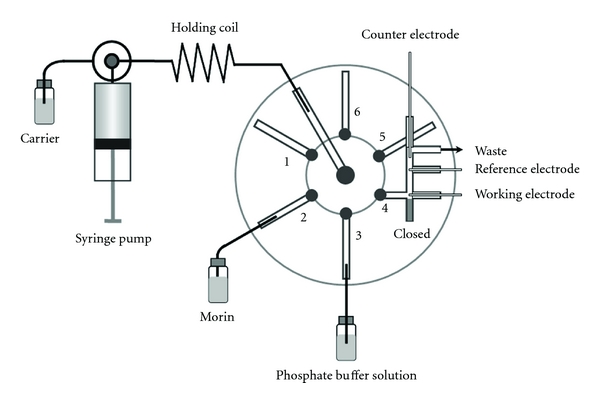
Schematic diagram of the SI-LOV manifold in conjunction with amperometry for the determination of morin.

The sequential injection lab-on-valve (SI-LOV) system composed of a FIAlab-3000 sequential injection system (FIAlab, Instruments, Bellevue, WA, USA), a high-precision 2500 *μ*L syringe pump (Cavro, Sunnyvale, CA, USA), and a six-position selector valve is shown schematically in Figure 2. Sample metering, mixing, dilution, reagent addition, incubation, separation, and detection can be executed in any desired sequence with LOV module consisting of all kinds of channels that are integrated with a multipurpose flow cell. The holding coil (HC) with capacities of 2.5 mL was made out of 0.8 mm i.d. PTFE tubing (Upchurch Scientific, Oak Harbor, WA, USA), which allowed chemical reaction and/or physical operations to take place. The instrument is controlled by FIAlab software for Windows 5.0 which allows for versatile and reliable sequencing of events comprising assay protocol and data collection. A homemade LOV unit incorporated an electrochemical flow cell (EFC) whose volume was equivalent to approximately 180 *μ*L with a three-electrode system, namely, a CPE modified by PVP as working electrode (WE), a platinum wire electrode as counter electrode (CE), and a Ag/AgCl electrode as reference electrode (RE). Moreover, the working electrode is placed in the canal adjacent to the sample inlet in order to have the best contact with the sample from EFC. The amperometric measurements were carried out with a LK2005 electrochemical workstation (LANLIKE Tianjin Chemistry and Electron High Technology Co. Ltd., China).

### 2.2. Chemicals

 All the reagents used were at least of analytical reagent grade and purchased from Sinopharm Chemical Reagent Co., Ltd. (Shanghai, China) unless otherwise stated. Double deionized water (18.2 MΩ cm) was used throughout the experiments. All the experiments were carried out at room temperature. Stock solution of 1 × 10^−2^ mol L^−1^ morin (Fluka) was prepared and kept at 4°C by dissolving 0.3022 g morin with water in 100 mL volumetric flask where a small quantity of 0.2 mol L^−1^ NaOH solution was added in order to promote dissolution. The working standard solutions were obtained by stepwise dilution using 0.05 mol L^−1^ phosphate buffer solution (PBS, pH 6.5). PBS (0.05 mol L^−1^, pH 6.5), and de-ionized water was used that acted as the supporting electrolyte and carrier solution, respectively. The pH value of buffer solution was adjusted by phosphoric acid solution or sodium hydroxide solution. PVP K-30 and graphite powder (CP) were used as electrode materials.

### 2.3. Fabrication and Pretreatment of Working Electrode

 The PVP-doped CPE was prepared by mixing 2.3 g graphite, 0.2 g PVP, and 500 *μ*L paraffin oil into a mortar to form a homogeneous mixture. And then, the mixture was pressed into a homemade cylindrical glass tube 6.0 mm i.d. with loading height of approximately 3.5 cm, which was connected with a copper wire (diameter: 1.0 mm), and the electrode surface was polished manually. The prepared PVP-CPE was activated at 100 mV/s over the potential range from −1.0 to 0.6 V using HAc-NaAc (0.1 mol L^−1^, pH 5.5) until stable cyclic voltammetry curves were obtained. All potentials were against Ag/AgCl in this work. Such pretreatment of the working electrodes allowed us to obtain a maximum sensitivity, and it was repeated after each regeneration of electrode. It is noted that the amount of paraffin oil should be restricted to a moderate amount since insufficient paraffin oil would be unfavorable for the formation of uniform mixture; excessive paraffin oil, however, would result in the decrease of conductivity of the electrode.

### 2.4. Sample Pretreatment

The tea samples were purchased from local market and treated as follows. The tea sample (about 1.0 g) was exactly weighed, and the morin was extracted with 30 mL of ether and acetone (14 : 1, v/v), vortexed for 5 min, then centrifuged for 20 min at 2000 rpm. The upper organic layer was transferred into another test tube and evaporated to dryness with N_2_. The residue was reconstituted in PBS, which was used for the assay of morin by the proposed methodology.

Mulberry leaves (*M. alba *L.) were purchased from local Chinese pharmacy store and finely pulverized by electric blender before the experiment. Twenty grams of powdered leaves was transferred into a beaker together with de-ionized water and a few drops of 0.2 mol L^−1^ NaOH solution for 24 hours. After being filtered through a filter paper, the extract was transferred to 100 mL conical beaker, and then the contents of morin were analyzed after appropriate dilution.

### 2.5. Operating Procedure

 The operation schematic diagram and the relative position of electrodes were illustrated in Figure 2. The central channel was connected to 2500 *μ*L high-precision syringe pump via HC. Port 4 acted as a detection port where an EFC was integrated while ports 1 and 5 and 6 were used as reserved channels. The assay protocol comprised the following steps. First, 600 *μ*L double-deionized water was drawn from the reservoir to clean all the lines and remove possible air bubbles. Then, 500 *μ*L of PBS and 200 *μ*L of morin solution were aspirated from the ports 3 and 2 sequentially at a flow rate of 50 *μ*L s^−1^ into the HC, where the different zones were stacked and stored. Thereafter, the mixed sample/reagent zones were propelled forward through the EFC at the flow rate of 30 *μ*L s^−1^ via port 4; at the same time, the resulting current for amperometric measurement was recorded when the external potential difference of 0.6 V was applied. Finally, the system was cleaned by pumping water through the flow cell.

## 3. Results and Discussion

### 3.1. Electrochemical Activity of Morin

**Figure 3:**
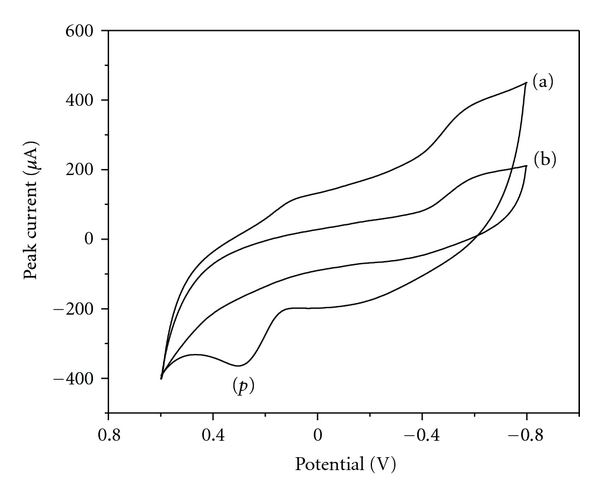
Cyclic voltammograms obtained at bare CPE (b) and PVP/CPE (a) surfaces using 1.0 × 10^−5^ mol L^−1^ morin solution in 0.05 mol L^−1^ PBS (pH 6.5).

The electrochemical characteristics of morin were studied by cyclic voltammetry (CV) using 1.0 × 10^−5^ mol L^−1^ morin solution in 0.05 mol L^−1^ PBS (pH 6.5). The electrochemical responses at bare CPE (b) and PVP/CPE (a) surfaces were presented in Figure 3. It was suggested that no peak was obtained at bare CPE. However, when PVP/CPE was applied, a well-defined oxidation peak (p) appeared at ca. 0.28 V with the anodic sweep from −0.8 to 0.6 V, which was related to the oxidation of morin. Meanwhile, a corresponding small reduction peak was observed in the reverse scan, which was related to the oxidation products of morin. The very low reduction peak currents indicate a low degree of reversibility of morin oxidation. As a nonionic polymer with amphiphilic character, PVP contains a carbonic backbone which pyrrolidine rings are attached to. A segment has the following chemical composition: C_6_H_9_NO, which corresponds to the overall composition of the polymer. PVP doped in CPE surface could effectively facilitate the adsorption of morin on the electrode surface and exhibit high accumulation efficiency, which would lead to the enhancement of current responses of morin. This was caused by the fact that PVP had the ability to form hydrogen bonds between imide in the center of polymer and hydroxyl group of morin [29]. During the following successive cyclic sweeps, the oxidation peak of morin decreases gradually. It may be caused by the fact that the electron transfer was blocked by the adsorption of reaction products resulting in the decrease of reaction sites on electrode surface.

### 3.2. Effects of Buffer Solution and pH

The supporting electrolyte that had a significant impact on the amperometric analysis was selected based on the effect of its pH on the peak height and peak shape. Under the condition of *ΔE* of 0.6 V, influences of various buffer systems on resulting currents were tested using 1.0 × 10^−5^ mol L^−1^ morin standard solution, *namely,* 0.05 mol L^−1^ Na_2_HPO_4_-KH_2_PO_4_, 0.05 mol L^−1^ Tris-HCl, and 0.1 mol L^−1^ HAc-NaAc. The results showed that the current response of morin in phosphate buffer solution was significantly higher than the other two kinds of mediums along with well-shaped peaks and stable current signals. As a result, PBS was chosen as the reaction medium. A more detailed study of the effect of pH on the amperometric response was performed using PBS (0.05 mol L^−1^) over the pH level of 3.5–8.5. It can be seen that the peak height increased with the pH value of PBS up to about 6.5, above which it decreased, and at which a maximum signal was obtained. Therefore, phosphate buffer solution of pH 6.5 was used throughout the experiments.

Furthermore, the influence of pH of PBS on peak potential was studied by CV over the pH range of 4.5–8.0. It was suggested that the oxidation peak potential shifted negatively with increasing solution pH, obeying the equation *E*_p_(V) = 0.6799 − 0.0613 pH with *R* ^2^ = 0.9949. The slope of 61.3 mV pH^−1^ was obtained, which suggested that the number of protons and electrons involved in the oxidation of morin was equal. In addition, the current intensity of the oxidation peak p decreased obviously with increase of solution pH. This may be due to the increasing deprotonation of OH groups in morin with increasing pH.

### 3.3. Electrode Process of Morin at PVP/CPE

**Figure 4:**
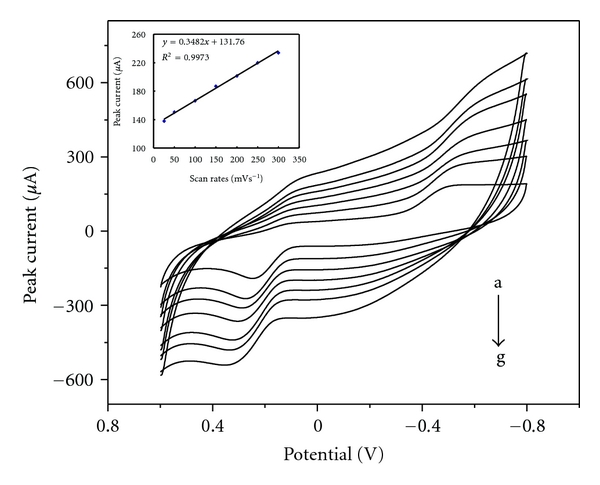
Cyclic voltammograms obtained at PVP/CPE in 0.05 mol L^−1^ PBS with various scan rates. The scan rates were 25, 50, 100, 150, 200, 250, 300 mV/s from a to g. Inset: plot of the peak current as a function of the scan rate.

The current responses of morin would be influenced by scan rate, and corresponding electrochemical parameters could be deduced from the relationship between scan rate of potential sweep and current responses of morin. The dependence of oxidation peak current of 1.0 × 10^−5^ mol L^−1^ morin on scan rate at PVP/CPE was illustrated in Figure 4. The oxidation peak current varied linearly with the scan rates (*ν*) over the range of 25 to 300 mV/s. A linear relationship between the peak current and the scan rate was found as *I*_p_(*μ*A) = 0.3482*v* + 131.76 (*R* ^2^ = 0.9973), suggesting that the electrochemical reaction process of morin at the PVP/CPE was an adsorption-controlled process.

### 3.4. Optimization of Percentage Composition of PVP

It is noted that the PVP/CPE can remarkably improve the oxidation peak current of morin. However, further studies showed that the amount of PVP contained in the doped CPE also affects the electrochemical response to morin. The influence of PVP amount on the oxidation peak current of morin was examined within the range of 2–14%. As the content of PVP is gradually improving, the oxidation peak current also increased correspondingly and reached its maximum at 8% of the compound electrode material, above which it decreased. As the amount of PVP is gradually rising, more active sites were obtained so that the oxidation peak current greatly increased. With further improvement of the content of PVP, however, the conductivity of PVP/CPE lowered, which clogged the electron transfer of morin and enhanced the background current. Thus, the oxidation peak current of morin contrarily decreased when excessive higher content of PVP was applied. Moreover, high PVP ratio in the composition could lead to its leaching from the electrode because of its solubility in water and other polar solvents. As a result, the best mass content of PVP was found to be 8%.

### 3.5. Optimal Working Potential

 The potential difference (Δ*E*) appeared to be a predominant parameter in amperometric measurement, which had an influence on sensitivity, selectivity, and peak shape. The influence of the Δ*E* on amperometric signal magnitude was investigated within the range of 0.1–1.0 V in 1.0 × 10^−5^ mol L^−1^ morin solution. With increasing *ΔE*, the current response was improved along with larger background current. However, when higher Δ*E* was applied above 0.6 V, the poor-quality peak shape was obtained with unstable baseline. This could be explained that the increasing of the applied Δ*E* leads to the increase of sensitivity accompanying the decrease of both selectivity and S/N ratio, meaning that possible interference of undesirable species presented in the measuring sample could be suppressed at the lower potential. With Δ*E* of 0.6 V, the well-shaped peak and more stable baseline were obtained. Therefore, imposed Δ*E* of 0.6 V was favored as a compromise between the sensitivity and selectivity for the further experiment.

With regard to amperometric detection mode, the background current which is mainly caused by other introduced electroactive substances, is more faradic current than charging current since the working potential difference is fixed at a constant value [30, 31].

### 3.6. Optimization of LOV Parameters

 The optimization of LOV parameters, *namely,* sample volume and flow rate, was carried out by the univariate method, with the aim to achieve a compromise between the peak height, sample throughput, and reproducibility. Since there was no homogeneous reaction in the proposed method, some conditions were not considered essential, such as reaction time, temperature, and the concentration of reagents.

It was found that the injection volume had a strong effect on current response in peak height and peak width, which varied from 50 to 350 *μ*L. The peak height and peak width increased with the sample volume up to 150 *μ*L, above which it leveled off, demonstrating the improvement of detection sensitivity by increasing the sample volume within a certain range. No significant differences were observed above 150 *μ*L except for a decrease in the sample throughput. This phenomenon could be attributed to the fact that the amount of reactant was increased with the increment of the injection volume within a certain range, resulting in an increase of the peak height. However, when an excessive amount of reagent zone was introduced into the EFC, the morin sample zone could only disperse/penetrate into a certain length of it, thus leaving a part of the zone with pure reagent, which contributed very little to the reaction in fact, and thus an unchanged signal was obtained. Therefore, a 200 *μ*L sample volume was assumed as the optimum with suitable peak width.

The flow rate was investigated taking into account the change in signal intensity as well as the peak shape and reproducibility, on which the sample throughput was strongly dependent. The signal intensity increased with the flow rate from 15 to 30 *μ*L s^−1^ and reached a maximum at the flow rate of 30 *μ*L/s; thereafter it decreased. The extent of dispersion is one of the most important factors affecting the reaction and the sensitivity of the detection system concerning the adjacent sample/reagent zones in a flow channel. When lower flow rate was implemented, a broader peak and small peak height were obtained in that the higher degree of dispersion of the sample zone into the carrier stream would result in the decrease of the concentration of the detectable sample. Higher flow rate, on the other hand, would lead to an unstable signal with poor reproducibility due to lack of adequate reaction time. It proved that the maximal peak height was achieved with satisfactory sample throughput when the flow rate of 30 *μ*L/s was applied. Hence, 30 *μ*L/s was chosen as the optimum flow rate.

### 3.7. Influence of Interferences

 The tolerance of the analytical method to foreign species which could be found in real samples containing morin was investigated by means of using standard solutions of 5.0 × 10^−6^ mol/L morin and adding various concentrations of the interfering substances. The tolerable concentration of each interfering compound was taken as the measured signal variation ±5.0% relative error, and the tolerable ratio of each foreign species was the following: 1000-fold for Na^+^, K^+^, and PO_4_^3−^; 800-fold for Cl^−^, Ac^−^, and Mg^2+^; 500-fold for SO_4_^2−^, NH_4_^+^, and Ca^2+^; 300-fold for NO_3_^−^, I^−^, Mn^2+^, Zn^2+^, and salicylic acid; 200-fold for Bi^3+^, Cu^2+^, Cr^3+^, Sn^2+^, gallic acid, catechol, and ethanol; 100-fold for Ni^2+^, Co^2+^, thiamine hydrochloride, tyrosine; 50-fold for uric acid, and phenylalanine; 30-fold for riboflavin and ascorbic acid. In addition, several substances, such as catechins, thearubigins, and theaflavins with molecular structures similar to morin also have no evident disturbance in the analysis of real samples, which proved that LOV-amperometric technique for the determination of morin in real samples is feasible with good anti-interference ability.

### 3.8. Analytical Characteristics

**Figure 5:**
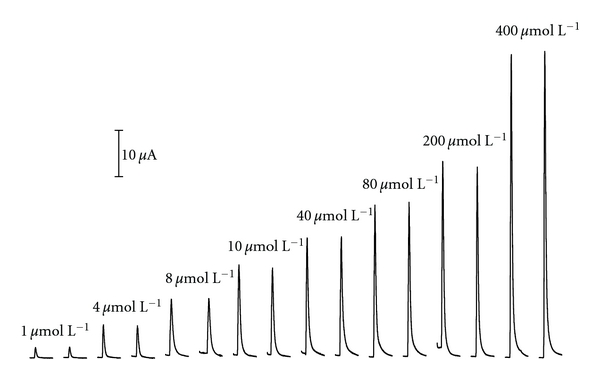
The amperometric current response with increasing concentrations of morin. *ΔE* of 0.6 V was applied. Sample volume was 200 *μ*L with the flow rate of 30 *μ*L s^−1^.

The analytical performance of the LOV procedure was evaluated by means of the application range, reproducibility, limit of detection (LOD), limit of quantitation (LOQ), and sample throughput. Under the selected experimental conditions above described, a calibration graph of the peak height versus concentrations of morin was obtained, which was described in Figure 5. The calibration graph was equipped with two linear sections. The first linear regression equation was *I*_p_ = 2.4366*C* − 0.7555 (*I*_p_: *μ*A, *C*: *μ*mol L^−1^) within the range of 1.0–10.0 *μ*mol L^−1^ of morin with a correlation coefficient of 0.9953. And the other one was *I*_p_ = 0.1456*C* + 24.973 (*I*_p_: *μ*A, *C*: *μ*mol L^−1^) within the range of 10.0–400.0 *μ*mol L^−1^) of morin with a correlation coefficient of 0.9959. The present method provided sufficient sensitivity as reflected by the values of LOD and LOQ. The calculation of LOD/LOQ for morin was yielded by utilizing LOD = 3 *σ*/*s* and LOQ = 10 *σ*/*s*, where *σ* is the standard deviation of the blank signals and *s* is the slope of the linear calibration graph. And the resulting concentrations at LOD and LOQ levels were 0.19 and 0.63 *μ*mol L^−1^, respectively. The RSDs of the proposed procedure determined by 11 replicates of samples at the levels of 5.0 and 40.0 *μ*mol L^−1^ were 3.1% and 3.7%, respectively. And the sample throughput of 45 h^−1^ was achieved.

The proposed method shows a lower LOD, a comparable concentration range of calibration curve, and a suitable sampling frequency, which have demonstrated the feasibility of the LOV platform with amperometric detection for the determination of morin. The low reagents/samples consumption (200 *μ*L of sample volume for each analysis) and high sample throughput (45 samples per hour) with good precision (RSDs = 3.1% and 3.7%, *n* = 11) rank the developed method competitive for routine analysis. The present work introduces the SI-LOV technique in connection with amperometric assay and highlights advantages of miniaturization: reduced sample/reagent consumption and reduction of effluent waste, small size of the apparatus that makes it portable, and advantages of programmable flow.

The effectiveness of PVP/CPE was demonstrated by a long-term stability study of its surface activity. Under the selected conditions, when the PVP/CPE electrode was applied to the analysis of 5.0 × 10^−6^ mol L^−1^ morin for 30 repetitive injections, the RSD of 3.3% was obtained, which indicated that the electrode did not had any problems or blocked from the adsorption of reaction products. What is more, all the manipulations were carried out in a closed system, which could greatly reduce the contaminations of electrodes. In a word, a much longer useful working period for PVP/CPE was achieved without significant variation in the response performance neither in the requirement of special conditioning treatments. The general good characteristics extended its use as a detector in the LOV system.

### 3.9. Application to the Practical Sample

 The present work introduces the lab-on-valve technique in connection with amperometric measurement, documents advantages of using programmable flow, and outlines means for miniaturization of assays. The feasibility of the established system was demonstrated by morin detection in tea and mulberry leaves (*M. alba *L.). Meanwhile, the spiked recovery experiments were performed with satisfactory results (Table 1), suggesting that the proposed method is feasible and effective with high accuracy for the determination of morin. Due to a matrix effect present in the sample in an unpredictable extent, quantification was achieved by the standard addition method.

## 4. Conclusions

A robust and straightforward automated procedure for the determination of morin in real sample is developed as an alternative analytical technique to other more costly and complicated procedure. With the use of programmable flow, advantages of LOV technique are the small size of the instrument and unprecedented reagent economy, which enriches the apparatus of miniaturization and automatization, broadening its range of application in electrochemical scope. The PVP/CPE integrated to EFC is easy to construct in laboratory and enables the implementation of easy regeneration accomplished by means of a quick mechanical renewal of the electrode surface layer. The PVP/CPE exhibited an excellent electrochemical activity towards the oxidation of morin in PBS, and the electrochemical characteristics of morin oxidation peak were well investigated. PVP could effectively facilitate the adsorption of morin on surface of the modified electrode, which led to the enhancement of the oxidation peak of morin. So above advantages make LOV-amperometric analytical platform a solid candidate for monitoring morin in real sample by the application of PVP/CPE.

## Figures and Tables

**Table 1:** Sequential injection lab-on-valve format hyphenated with amperometry based on PVP/CPE with applications to the analysis of morin in real samples^a^.

Samples	Original	Spiked	Total found	Recovery (%)	RSD (%, *n* = 3)
	(10^−5^ mol L^−1^)	(10^−5^ mol L^−1^)	(10^−5^ mol L^−1^)		
Tea	1.76 ± 0.28	2.00	3.70 ± 0.35	97.0 ± 0.6	2.6
		4.00	5.86 ± 0.44	102.5 ± 0.9	2.7

Mulberry leaves	5.13 ± 0.27	6.00	11.27 ± 0.46	102.3 ± 1.1	3.1
		8.00	13.42 ± 0.53	103.6 ± 1.2	3.6

^a^The results were obtained at 95% confidence level.
